# 2,6-Dimethyl­pyridinium bromide

**DOI:** 10.1107/S1600536812039578

**Published:** 2012-09-22

**Authors:** Salim F. Haddad, Basem F. Ali, Rawhi Al-Far

**Affiliations:** a Department of Chemistry, The University of Jordan, Amman 11942, Jordan; b Department of Chemistry, Al al-Bayt University, Mafraq 25113, Jordan; c Faculty of Science and IT, Al-Balqa’a Applied University, Salt, Jordan; d Qassim University, Faculty of Science, Chemistry Department, Qassim, Saudi Arabia

## Abstract

The asymmetric unit of the title salt, C_7_H_10_N^+^·Br^−^, comprises two 2,6-dimethyl­pyridinium cations and two bromide anions. One cation and one anion are situated in general positions, while the other cation and the other anion lie on a crystallographic mirror plane parallel to (010). Each pair of ions inter­act *via* N—H⋯Br and C—H⋯Br hydrogen bonding, generating motifs depending on the cation and anion involved. Thus, the cation and the anion on the mirror plane generate infinite chains along the *c* axis, while the other ionic pair leads to sheets parallel to the *ac* plane. In the overall crystal packing, both motifs alternate along the *b* axis, with a single layer of the chain motif sandwiched between two double layers of the sheet motif. The sheets and chains are further connected *via* aryl π–π inter­actions [centroid–centroid distances = 3.690 (2) and 3.714 (2) Å], giving a three-dimensional network.

## Related literature

For background on the structural importance of noncovalent inter­actions, see: Desiraju (1997 ▶); Hunter (1994 ▶); Allen *et al.* (1997 ▶); Dolling *et al.* (2001 ▶); Panunto *et al.* (1987 ▶); Robinson *et al.* (2000 ▶). For related geometric parameters, see: Allen *et al.* (1987 ▶); Ahmadi *et al.* (2008 ▶); Amani *et al.* (2008 ▶); Jin *et al.* (2000 ▶, 2003 ▶, 2006 ▶); Nuss *et al.* (2005 ▶); Pan *et al.* (2001 ▶). For related literature on ar­yl⋯aryl inter­actions, see: Gould *et al.* (1985 ▶); Hunter & Sanders (1990 ▶); Hunter (1994 ▶); Singh & Thornton (1990 ▶).
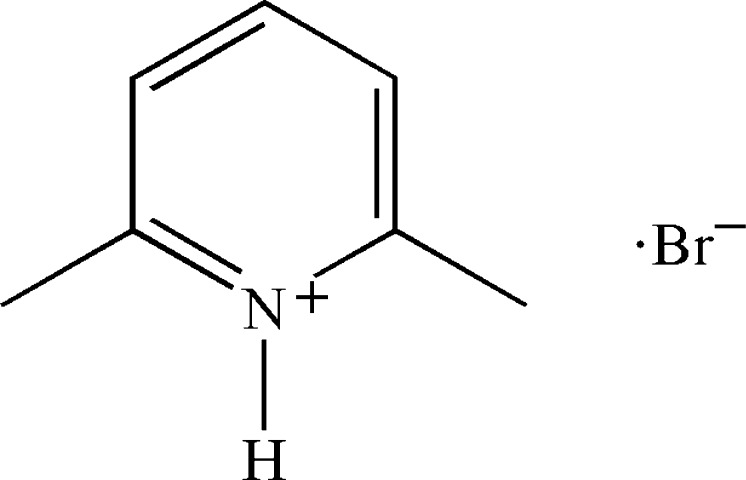


## Experimental

### 

#### Crystal data

C_7_H_10_N^+^·Br^−^
*M* *_r_* = 188.06
Orthorhombic, 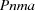

*a* = 15.0788 (13) Å
*b* = 20.432 (3) Å
*c* = 7.8456 (7) Å
*V* = 2417.2 (5) Å^3^
*Z* = 12
Mo *K*α radiation
μ = 5.02 mm^−1^
*T* = 293 K
0.30 × 0.25 × 0.20 mm

#### Data collection

Agilent Xcalibur Eos diffractometer
Absorption correction: multi-scan (*CrysAlis PRO*; Agilent, 2011 ▶) *T*_min_ = 0.242, *T*_max_ = 0.367
6863 measured reflections
2194 independent reflections
1708 reflections with *I* > 2σ(*I*)
*R*_int_ = 0.036

#### Refinement

*R*[*F* ^2^ > 2σ(*F* ^2^)] = 0.040
*wR*(*F* ^2^) = 0.102
*S* = 1.03
2194 reflections
140 parameters
H-atom parameters constrained
Δρ_max_ = 0.46 e Å^−3^
Δρ_min_ = −0.43 e Å^−3^

### 

Data collection: *CrysAlis PRO* (Agilent, 2011 ▶); cell refinement: *CrysAlis PRO*; data reduction: *CrysAlis PRO*; program(s) used to solve structure: *SHELXS97* (Sheldrick, 2008 ▶); program(s) used to refine structure: *SHELXL97*; molecular graphics: *SHELXTL* and *OLEX2* (Dolomanov *et al.*, 2009 ▶); software used to prepare material for publication: *SHELXTL*.

## Supplementary Material

Crystal structure: contains datablock(s) I, global. DOI: 10.1107/S1600536812039578/lr2077sup1.cif

Structure factors: contains datablock(s) I. DOI: 10.1107/S1600536812039578/lr2077Isup2.hkl

Supplementary material file. DOI: 10.1107/S1600536812039578/lr2077Isup3.cml

Additional supplementary materials:  crystallographic information; 3D view; checkCIF report

## Figures and Tables

**Table 1:** Hydrogen-bond geometry (Å, °)

*D*—H⋯*A*	*D*—H	H⋯*A*	*D*⋯*A*	*D*—H⋯*A*
N2—H2*A*⋯Br1	0.86	2.34	3.199 (5)	180
N1—H1*A*⋯Br2	0.86	2.32	3.182 (4)	179
C4—H4*A*⋯Br2^i^	0.93	2.83	3.722 (5)	161
C2—H2*B*⋯Br2^ii^	0.93	2.76	3.664 (5)	163
C9—H9*A*⋯Br1^iii^	0.93	2.97	3.842 (6)	157

Symmetry codes: (i) 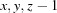; (ii) 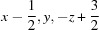; (iii) 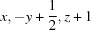.

**Fig. 1.**
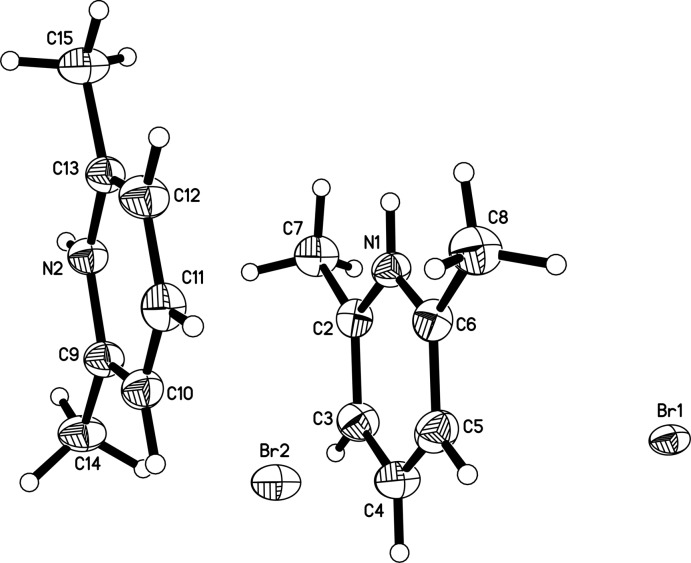
The asymmetric unit of the title compound. Displacement ellipsoids are drawn at the 30% probability level.

**Fig. 2.**
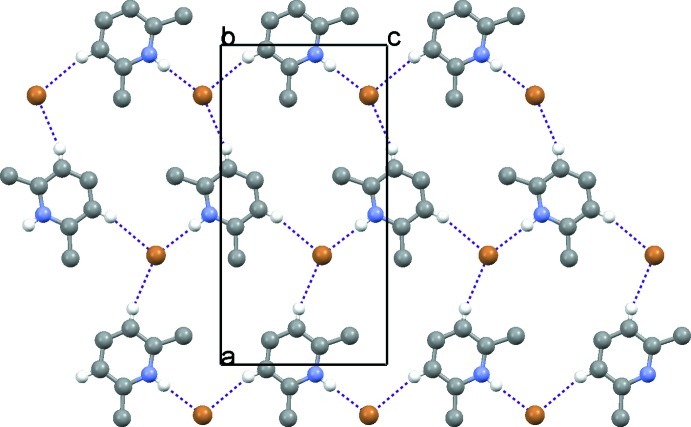
Partial view of the crystal packing showing the sheet motifs parallel to (010). Hydrogen bonds are shown as dashed lines.

**Fig. 3.**
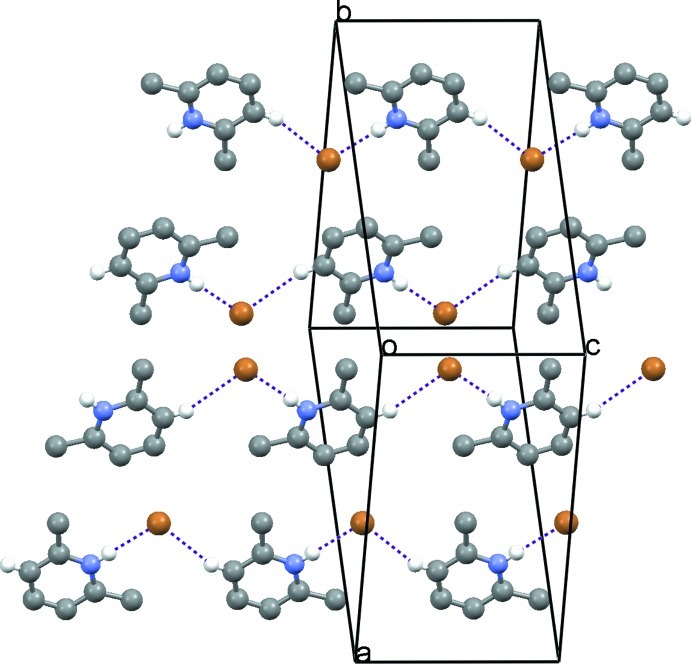
Another view of the crystal packing showing the infinite chains runnig along the c axis, with the involved N–H···Br and C–H···Br interactions showed as dashed lines.

**Fig. 4.**
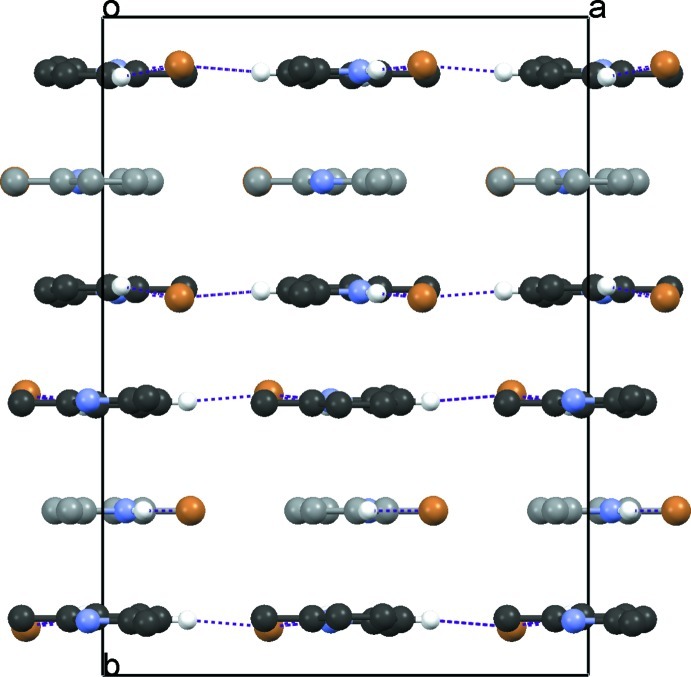
A view of the overall crystal packing showing single layers of the chain motif sandwiched between double layers of the sheet type motif.

## supplementary crystallographic information

### Comment

Non-covalent interactions play an important role in organizing structural units in both natural and artificial systems (Desiraju, 1997). They exercise important effects on the organization and properties of many materials in areas such as biology (Hunter, 1994), crystal engineering (Allen *et al.*, 1997, Dolling *et al.*, 2001) and material science (Panunto *et al.*, 1987, Robinson *et al.*, 2000). The interactions governing the crystal organization are expected to affect the packing and then the specific properties of solids. We herein report the structure of the salt, 2,6-dimethylpyridinium with the bromide anion, along with its crystal packing.

The asymmetric unit of title salt comprises two 2,6-dimethylpyridinium (hereafter 2,6-dmpH) cation: A (containing N1/C1/C2/C3/C4/C5) and B (containing N2/C8/C9/C10/C11/C12), and two bromide anions (Br1 and Br2), Fig. 1. Cation A as well as the anion Br2 are situated in general positions; while cation B, as well as the Br1 bromide anion are lying on a mirror plane parallel to (010). Both cations having almost identical geometrical parameters, and fall within the range expected (Allen *et al.*, 1987, Ahmadi *et al.*, 2008, Amani *et al.*, 2008, Jin *et al.*, 2003, Nuss *et al.*, 2005, Jin *et al.*, 2006). When compared to pyridine, the C–N–C angles (124.1 (3) and 123.7 (4)° in cations A and B, respectively) in the title compound are widened. This is in good agreement with other reported salts of 2,6-dmpH with different anions, such as the dichromate [124.6 (3)°; (Jin *et al.*, 2006)], the chloride [124.1 (1)°; (Nuss *et al.*, 2005)], the nitrate [124.90 (13)°; (Jin *et al.*, 2003)], the hydrogen phthalate [128.83 (2)°; (Jin *et al.*, 2000)] and the hydrogen fumarate [123.9 (2)°; (Pan *et al.*, 2001)].

The crystal structure of title salt present a supramolecular network, where a complex strong hydrogen-bonding scheme operates between the cations and the anions (Table 1). The 2,6-dmpH (N and C atoms) act as donors, with the Br atoms the acceptors. The supramolecular hydrogen-bonding N–H···Br and C–H···Br synthons are shown in Figures 2 and 3. These hydrogen bonds connect the cations type A and Br2 anions into sheets parallel to the *ac* plane, Fig. 2. Within each sheet the cations and anions might be considered as packed in a pseudo three-fold arrangement (each cation is connected to three surrounding anions and each anion is connected to three surrounding cations) that extends in the *ac* plane, Fig. 2. On the other hand, cations type B with Br1 anions are forming infinite chains along the *c* crystallographic axis, *via* N2-H2A···Br1···H9-C9 hydrogen bonding, in which each Br1 anions linking two cations through double H···Br···H interactions, Fig. 3.

The overall packing can be describes as of the *sandwich* type, in which double layers of the sheet type motif formed by cations A and Br2 anions moieties, alternate with single layers of the chains motif type formed by cations B and Br1 anions moieties down the *b* axis, Fig. 4. Both packing motifs interact through *offset-face-to-face* aryl···aryl interactions with centroids distances of 3.690 Å for A(C_g_)···B(C_g_) and 3.712 Å for A(Cg)···A(C_g_) (1 - *x*, 1 - *y*, -*z*), giving a three-dimensional network. These separation distances are in accordance with those of calculated and the experimentally observed stacked (*offset-face-to-face*) interaction modes (Gould *et al.*, 1985, Hunter & Sanders, 1990, Hunter, 1994, Singh & Thornton, 1990).

### Experimental

In an attempt to crystallize a tetrahalomercurate with the 2,6-dimethylpyridinium cation, the title compound crystallized instead. To a warm solution of 2,6-Dimethylpyridine (1 mmol) and 1 ml 60% HBr dissolved in 95% EtOH (10 ml), a hot solution of HgCl_2_ (1 mmol) dissolved in 95% EtOH (10 ml) was added. The resulting mixture was then treated with Br_2_ (2–3 ml) and refluxed for 3 hrs. The resulting mixture was left undisturbed to evaporate at room temperature whereupon colorless block crystals are formed after three days.

### Refinement

All hydrogen atoms constrained and assigned isotropic thermal parameters of 1.2 times that of the riding atoms (1.5 for methyl). Largest diff. peak and hole were 0.478 and -0.478 e.Å^-3^ with largest peak 1.035 Å from Br1.

### Crystal data

**Table:**

C_7_H_10_N^+^·Br^−^	*F*(000) = 1128
*M**_r_* = 188.06	*D*_x_ = 1.550 Mg m^−^^3^
Orthorhombic, *P**n**m**a*	Mo *K*α radiation, λ = 0.71073 Å
Hall symbol: -P 2ac 2n	Cell parameters from 1341 reflections
*a* = 15.0788 (13) Å	θ = 3.0–29.3°
*b* = 20.432 (3) Å	µ = 5.02 mm^−^^1^
*c* = 7.8456 (7) Å	*T* = 293 K
*V* = 2417.2 (5) Å^3^	Block, colourless
*Z* = 12	0.30 × 0.25 × 0.20 mm

### Data collection

**Table:**

Agilent Xcalibur Eos diffractometer	2194 independent reflections
Radiation source: Enhance (Mo) X-ray Source	1708 reflections with *I* > 2σ(*I*)
Graphite monochromator	*R*_int_ = 0.036
Detector resolution: 16.0534 pixels mm^-1^	θ_max_ = 25.0°, θ_min_ = 3.4°
ω scans	*h* = −17→11
Absorption correction: multi-scan (*CrysAlis PRO*; Agilent, 2011)	*k* = −23→24
*T*_min_ = 0.242, *T*_max_ = 0.367	*l* = −9→7
6863 measured reflections	

### Refinement

**Table:**

Refinement on *F*^2^	Primary atom site location: structure-invariant direct methods
Least-squares matrix: full	Secondary atom site location: difference Fourier map
*R*[*F*^2^ > 2σ(*F*^2^)] = 0.040	Hydrogen site location: inferred from neighbouring sites
*wR*(*F*^2^) = 0.102	H-atom parameters constrained
*S* = 1.03	*w* = 1/[σ^2^(*F*_o_^2^) + (0.0499*P*)^2^ + 0.7328*P*] where *P* = (*F*_o_^2^ + 2*F*_c_^2^)/3
2194 reflections	(Δ/σ)_max_ = 0.001
140 parameters	Δρ_max_ = 0.46 e Å^−^^3^
0 restraints	Δρ_min_ = −0.43 e Å^−^^3^

### Special details

**Table:**

Geometry. All e.s.d.'s (except the e.s.d. in the dihedral angle between two l.s. planes) are estimated using the full covariance matrix. The cell e.s.d.'s are taken into account individually in the estimation of e.s.d.'s in distances, angles and torsion angles; correlations between e.s.d.'s in cell parameters are only used when they are defined by crystal symmetry. An approximate (isotropic) treatment of cell e.s.d.'s is used for estimating e.s.d.'s involving l.s. planes.
Refinement. Refinement of *F*^2^ against ALL reflections. The weighted *R*-factor *wR* and goodness of fit *S* are based on *F*^2^, conventional *R*-factors *R* are based on *F*, with *F* set to zero for negative *F*^2^. The threshold expression of *F*^2^ > σ(*F*^2^) is used only for calculating *R*-factors(gt) *etc*. and is not relevant to the choice of reflections for refinement. *R*-factors based on *F*^2^ are statistically about twice as large as those based on *F*, and *R*- factors based on ALL data will be even larger.

### Fractional atomic coordinates and isotropic or equivalent isotropic displacement parameters (Å^2^)

**Table:**

	*x*	*y*	*z*	*U*_iso_*/*U*_eq_	Occ. (<1)
N1	0.0302 (2)	0.08268 (13)	0.5688 (4)	0.0385 (7)	
H1A	0.0643	0.0802	0.6564	0.046*	
C1	−0.0586 (3)	0.08263 (16)	0.5951 (5)	0.0397 (9)	
C2	−0.1132 (3)	0.08487 (18)	0.4561 (6)	0.0477 (10)	
H2B	−0.1744	0.0839	0.4697	0.057*	
C3	−0.0765 (3)	0.08853 (18)	0.2958 (6)	0.0511 (11)	
H3A	−0.1134	0.0908	0.2011	0.061*	
C4	0.0140 (3)	0.08893 (17)	0.2736 (5)	0.0503 (10)	
H4A	0.0380	0.0910	0.1646	0.060*	
C5	0.0685 (3)	0.08625 (17)	0.4130 (5)	0.0419 (10)	
C6	−0.0902 (3)	0.07923 (18)	0.7754 (5)	0.0519 (11)	
H6A	−0.0420	0.0892	0.8510	0.078*	
H6B	−0.1117	0.0360	0.7990	0.078*	
H6C	−0.1372	0.1103	0.7920	0.078*	
C7	0.1671 (3)	0.08641 (19)	0.4050 (6)	0.0568 (12)	
H7A	0.1896	0.1233	0.4675	0.085*	
H7B	0.1857	0.0894	0.2883	0.085*	
H7C	0.1895	0.0467	0.4541	0.085*	
N2	0.4525 (3)	0.2500	0.7490 (5)	0.0406 (10)	
H2A	0.4164	0.2500	0.6640	0.049*	
C8	0.4177 (4)	0.2500	0.9087 (7)	0.0403 (13)	
C9	0.4755 (4)	0.2500	1.0449 (7)	0.0450 (13)	
H9A	0.4542	0.2500	1.1561	0.054*	
C10	0.5655 (4)	0.2500	1.0135 (7)	0.0480 (14)	
H10A	0.6049	0.2500	1.1047	0.058*	
C11	0.5977 (4)	0.2500	0.8509 (8)	0.0502 (15)	
H11A	0.6585	0.2500	0.8319	0.060*	
C12	0.5402 (3)	0.2500	0.7161 (7)	0.0397 (12)	
C13	0.3189 (4)	0.2500	0.9253 (8)	0.0522 (15)	
H13A	0.2969	0.2063	0.9096	0.078*	0.50
H13B	0.2937	0.2783	0.8404	0.078*	0.50
H13C	0.3026	0.2654	1.0366	0.078*	0.50
C14	0.5682 (4)	0.2500	0.5325 (7)	0.0578 (16)	
H14A	0.5901	0.2926	0.5023	0.087*	0.50
H14B	0.5182	0.2393	0.4619	0.087*	0.50
H14C	0.6141	0.2181	0.5158	0.087*	0.50
Br1	0.31927 (4)	0.2500	0.43172 (8)	0.0566 (2)	
Br2	0.15804 (3)	0.07357 (3)	0.89056 (6)	0.0627 (2)	

### Atomic displacement parameters (Å^2^)

**Table:**

	*U*^11^	*U*^22^	*U*^33^	*U*^12^	*U*^13^	*U*^23^
N1	0.0341 (18)	0.0458 (17)	0.0357 (17)	−0.0006 (14)	−0.0011 (14)	−0.0024 (15)
C1	0.036 (2)	0.039 (2)	0.044 (2)	−0.0048 (17)	0.0076 (19)	0.0001 (17)
C2	0.030 (2)	0.057 (2)	0.057 (3)	−0.0002 (18)	0.000 (2)	−0.004 (2)
C3	0.051 (3)	0.059 (2)	0.043 (2)	0.000 (2)	−0.008 (2)	0.001 (2)
C4	0.054 (3)	0.059 (2)	0.039 (2)	0.003 (2)	0.004 (2)	−0.002 (2)
C5	0.035 (2)	0.044 (2)	0.046 (2)	−0.0027 (17)	0.0079 (19)	−0.0013 (18)
C6	0.044 (2)	0.066 (3)	0.046 (2)	−0.003 (2)	0.010 (2)	0.003 (2)
C7	0.037 (2)	0.077 (3)	0.056 (3)	−0.002 (2)	0.009 (2)	0.001 (2)
N2	0.035 (2)	0.048 (2)	0.039 (3)	0.000	0.000 (2)	0.000
C8	0.035 (3)	0.046 (3)	0.040 (3)	0.000	0.003 (2)	0.000
C9	0.051 (3)	0.047 (3)	0.038 (3)	0.000	−0.002 (3)	0.000
C10	0.042 (3)	0.054 (3)	0.048 (3)	0.000	−0.012 (3)	0.000
C11	0.032 (3)	0.063 (4)	0.055 (4)	0.000	0.001 (3)	0.000
C12	0.034 (3)	0.042 (3)	0.043 (3)	0.000	0.002 (3)	0.000
C13	0.032 (3)	0.072 (4)	0.052 (4)	0.000	0.006 (3)	0.000
C14	0.048 (4)	0.078 (4)	0.048 (4)	0.000	0.010 (3)	0.000
Br1	0.0362 (3)	0.0925 (5)	0.0410 (3)	0.000	0.0003 (3)	0.000
Br2	0.0390 (3)	0.1034 (4)	0.0456 (3)	0.0040 (2)	−0.00588 (19)	−0.0051 (2)

### Geometric parameters (Å, º)

**Table:**

N1—C1	1.354 (5)	N2—C12	1.348 (6)
N1—C5	1.354 (5)	N2—C8	1.358 (6)
N1—H1A	0.8600	N2—H2A	0.8600
C1—C2	1.368 (6)	C8—C9	1.379 (7)
C1—C6	1.494 (5)	C8—C13	1.496 (7)
C2—C3	1.376 (6)	C9—C10	1.379 (8)
C2—H2B	0.9300	C9—H9A	0.9300
C3—C4	1.376 (6)	C10—C11	1.365 (8)
C3—H3A	0.9300	C10—H10A	0.9300
C4—C5	1.369 (6)	C11—C12	1.367 (7)
C4—H4A	0.9300	C11—H11A	0.9300
C5—C7	1.487 (6)	C12—C14	1.501 (7)
C6—H6A	0.9600	C13—H13A	0.9600
C6—H6B	0.9600	C13—H13B	0.9600
C6—H6C	0.9600	C13—H13C	0.9600
C7—H7A	0.9600	C14—H14A	0.9600
C7—H7B	0.9600	C14—H14B	0.9600
C7—H7C	0.9600	C14—H14C	0.9600
			
C1—N1—C5	124.0 (4)	C12—N2—C8	123.8 (5)
C1—N1—H1A	118.0	C12—N2—H2A	118.1
C5—N1—H1A	117.9	C8—N2—H2A	118.1
N1—C1—C2	118.3 (4)	N2—C8—C9	118.1 (5)
N1—C1—C6	117.4 (4)	N2—C8—C13	117.7 (5)
C2—C1—C6	124.4 (4)	C9—C8—C13	124.2 (5)
C1—C2—C3	119.2 (4)	C10—C9—C8	119.0 (5)
C1—C2—H2B	120.4	C10—C9—H9A	120.5
C3—C2—H2B	120.4	C8—C9—H9A	120.5
C4—C3—C2	121.0 (4)	C11—C10—C9	121.1 (5)
C4—C3—H3A	119.5	C11—C10—H10A	119.5
C2—C3—H3A	119.5	C9—C10—H10A	119.5
C5—C4—C3	119.6 (4)	C10—C11—C12	119.9 (5)
C5—C4—H4A	120.2	C10—C11—H11A	120.1
C3—C4—H4A	120.2	C12—C11—H11A	120.1
N1—C5—C4	117.8 (4)	N2—C12—C11	118.3 (5)
N1—C5—C7	117.7 (4)	N2—C12—C14	117.3 (5)
C4—C5—C7	124.5 (4)	C11—C12—C14	124.4 (5)
C1—C6—H6A	109.5	C8—C13—H13A	109.5
C1—C6—H6B	109.5	C8—C13—H13B	109.5
H6A—C6—H6B	109.5	H13A—C13—H13B	109.5
C1—C6—H6C	109.5	C8—C13—H13C	109.5
H6A—C6—H6C	109.5	H13A—C13—H13C	109.5
H6B—C6—H6C	109.5	H13B—C13—H13C	109.5
C5—C7—H7A	109.5	C12—C14—H14A	109.5
C5—C7—H7B	109.5	C12—C14—H14B	109.5
H7A—C7—H7B	109.5	H14A—C14—H14B	109.5
C5—C7—H7C	109.5	C12—C14—H14C	109.5
H7A—C7—H7C	109.5	H14A—C14—H14C	109.5
H7B—C7—H7C	109.5	H14B—C14—H14C	109.5
			
C5—N1—C1—C2	1.5 (5)	C12—N2—C8—C9	0.000 (2)
C5—N1—C1—C6	−179.3 (3)	C12—N2—C8—C13	180.000 (1)
N1—C1—C2—C3	−1.4 (5)	N2—C8—C9—C10	0.000 (2)
C6—C1—C2—C3	179.4 (4)	C13—C8—C9—C10	180.000 (1)
C1—C2—C3—C4	1.0 (6)	C8—C9—C10—C11	0.000 (2)
C2—C3—C4—C5	−0.6 (6)	C9—C10—C11—C12	0.000 (2)
C1—N1—C5—C4	−1.1 (5)	C8—N2—C12—C11	0.000 (1)
C1—N1—C5—C7	179.4 (3)	C8—N2—C12—C14	180.0
C3—C4—C5—N1	0.6 (5)	C10—C11—C12—N2	0.000 (1)
C3—C4—C5—C7	−180.0 (3)	C10—C11—C12—C14	180.000 (1)

### Hydrogen-bond geometry (Å, º)

**Table:**

*D*—H···*A*	*D*—H	H···*A*	*D*···*A*	*D*—H···*A*
N2—H2*A*···Br1	0.86	2.34	3.199 (5)	180
N1—H1*A*···Br2	0.86	2.32	3.182 (4)	179
C4—H4*A*···Br2^i^	0.93	2.83	3.722 (5)	161
C2—H2*B*···Br2^ii^	0.93	2.76	3.664 (5)	163
C9—H9*A*···Br1^iii^	0.93	2.97	3.842 (6)	157

Symmetry codes: (i) *x*, *y*, *z*−1; (ii) *x*−1/2, *y*, −*z*+3/2; (iii) *x*, −*y*+1/2, *z*+1.
